# Enteral Tube Feeding in Paediatric Mitochondrial Diseases

**DOI:** 10.1038/s41598-017-17256-7

**Published:** 2017-12-04

**Authors:** Han Som Choi, Young-Mock Lee

**Affiliations:** Department of Pediatrics, Gangnam Severance Hospital, Yonsei University College of Medicine, Seoul, Korea

## Abstract

We investigated the effects of enteral tube feeding in Korean children with mitochondrial diseases. We performed a retrospective chart review of 68 paediatric patients with mitochondrial diseases on enteral tube feeding at a tertiary referral centre. The outcome of enteral nutrition was evaluated by decrease in gastrointestinal (GI) symptoms, weight gain, and increase in developmental quotient (DQ) among patients with data available. Among 68 patients, 56 (82%) were on gastrostomy and 12 (18%) were on prolonged nasogastric (NG) tube feeding. Decrease of GI symptoms was present in 37 of 48 patients (77%). Weight gain was present in 18 of 64 patients (28%) and was more prominent in the gastrostomy group (n = 17/54, 32%). Increase in DQ was similar between the NG tube and gastrostomy groups (total n = 10/48, 21%). Complications occurred in 42% (n = 5/12) of the NG tube group and 64% (n = 36/56) of the gastrostomy group. They varied in range, from mild to severe. Most complications were minor; there were 5 cases (9%) requiring gastrostomy removal or additional procedure and 2 cases (4%) of gastrostomy-related morbidity. Our results show that in paediatric patients with mitochondrial diseases, enteral tube feeding could help enhance quality of life by relieving GI symptoms, ameliorate growth failure and enhance development.

## Introduction

Mitochondria are the hub of numerous metabolic pathways, including pyruvate oxidation, metabolism of basic nutrients, and generation of adenosine triphosphate (ATP)^1^. Disorders in any of these pathways cause mitochondrial diseases^1^. Studies have focused on defects in the respiratory chain complex, the major site of ATP production, due to its crucial role in life maintenance^1,2^. Defective ATP metabolism leads to insufficient energy supply, thus mitochondrial diseases mainly involve organs with relatively higher energy requirements, such as the brain, heart, and muscles^2^.

Mitochondrial diseases are prevalent in 10 to 15 cases per 100,000 persons^1^. They are heterogeneous and include diverse syndromes, but the most common manifestation is non-specific^2^. Neurodevelopmental regression and neurologic symptoms are key indicators of the diseases^2^. A study of 73 paediatric patients with the diseases showed 90% neurologic involvement during the median 12-year follow-up period and mortality rate of 46%^3^.

As mitochondrial diseases involve multiple organs, gastrointestinal (GI) involvement or nutritional deficit is common. GI involvement is reportedly present in 29%^3^ and 48%^4^ of patients with mitochondrial diseases, and failure to thrive is reportedly present in 52%^3,4^. Major GI symptoms include persistent vomiting, failure to thrive, and dysphagia^2^. Swallowing difficulties could inhibit sufficient oral intake and may result in aspiration or chest infection^5^. Failure to thrive and inadequate oral intake could inhibit long-term growth and nutrition^5^.

Enteral tube feeding is indicated in patients with neurologic diseases^6^. Nasogastric (NG) tube feeding is useful for 1 to 3 months, and gastrostomy is the appropriate option if feeding problems persist^7^. Research shows that gastrostomy benefits paediatric patients with neurodevelopmental diseases in terms of clinical progress and quality of life^5^. Complication rates of enteral tube feeding reportedly range from 10%^8^ to 80%^9^, with 5%^8^ to 20%^9^ of patients developing major complications.

As gastrostomy shows promising outcomes in the nutrition of neurologic patients, researchers have evaluated its effect in neurologic diseases such as cerebral palsy or Rett syndrome^10,11^. We have found no previous research on enteral tube feeding in patients with mitochondrial diseases. In this study, we report indications and outcomes of enteral feeding in 68 paediatric patients with mitochondrial diseases.

## Materials and Methods

### Patient selection and diagnostic evaluation

We performed a retrospective chart review of paediatric patients diagnosed with mitochondrial diseases in Gangnam Severance hospital, a tertiary medical referral centre in Seoul, Korea. Of 372 patients diagnosed with mitochondrial diseases, 68 patients began enteral tube feeding from July 2005 to June 2016.

Clinical characteristics of the patients were analysed by gender, disease onset, systemic involvement, and disease progress. Laboratory test results were obtained, including serum lactic acid. Muscle biopsies were performed surgically from the quadriceps muscle, and routine histological, immunohistochemical, and electron microscopic exams were conducted. Biochemical assays to evaluate mitochondrial respiratory chain enzyme activity were also performed, defining the enzyme to be defective when residual enzyme activity was below 10% of reference. Diagnosis of mitochondrial diseases was made based on biochemical, pathological, and clinical data as suggested by Bernier *et al*.^12^. The clinical diagnosis of syndromes was confirmed at an outpatient clinic or during admission. All procedures conducted were approved by the institutional review board of Gangnam Severance Hospital in Seoul, Korea. Informed consent were obtained and all methods were performed in accordance with the relevant guidelines and regulations of the board.

### Enteral tube feeding

We reviewed the patients’ age at the beginning of enteral tube feeding, interval between the onset of the disease and the start of enteral tube feeding, and GI symptoms leading to enteral tube feeding. Enteral tube feeding specified the use of NG tube or gastrostomy for nutritional support beyond the oesophagus, excluding oral nutritional supplements in enteral nutrition^6^. No patient in our study was on oral supplement. Prolonged NG feeding was defined as NG tube feeding for over four weeks due to GI symptoms related to the patients’ diseases. Patients who received tube feeding due to transient decline in general condition were excluded. Gastrostomy included percutaneous endoscopic gastrostomy (PEG) and surgical gastrostomy, either by laparoscopy or open surgery.

We discussed all the patients’ nutrition plan with the nutrition support team in the hospital. Patients fed on standard enteral formula with energy density approximating 1 kcal/ml. The composition of fat, carbohydrate and protein was not altered. As recent data supports bolus feeding as physiologic and standard^6^, we set all patients’ feeding end point as bolus feeding. Some patients went through transient continuous feeding for adaptation to enteral tube feeding, but all settled with bolus feeding.

### Assessment of outcomes and complications

Outcomes of enteral nutrition were evaluated by decrease in GI symptoms, weight gain, and increase in developmental quotients (DQ) among patients with data available. GI symptoms reflect the indications, or initiative causes, of enteral tube feeding. Weight gain represents the physical and nutritional effect of enteral tube feeding. DQ shows the developmental and systemic effect of enteral tube feeding. Progress of symptoms was assessed by chart review. We confirmed the patients’ decrease in GI symptoms if the patients or guardians reported decreased symptoms for at least two consecutive hospital visits following the start of enteral tube feeding. Patients’ weight was measured at each hospital visit in kilograms and converted to z-scores according to the Korean national growth chart^13^. We defined ‘weight gain’ as an increase in z-score after one year from the start of enteral tube feeding. DQ was evaluated by the Korean Infant and Child Developmental Test (KICDT)^14^ at every admission. It assesses five categories of gross motor, fine motor, language, cognitive-adaptive, and social-personal skills. We defined ‘increase in DQ’ as the increase in DQ at least three months after the initiation of enteral tube feeding.

We examined complications of enteral tube feeding with patients divided into NG tube and gastrostomy groups. Chart review revealed minor complications, such as abdominal pain and gastrostomy site granulations, and major complications involving gastrostomy removal or morbidity related to gastrostomy.

For statistical analysis of outcomes, we used SPSS version 23.0 (SPSS Inc., Chicago, IL, USA).

## Results

### Patient characteristics

Sixty-eight patients up to age 18 years old were diagnosed with mitochondrial diseases and started enteral tube feeding from July 2005 to June 2016 **(**Table 1 **)**. Mean age at the onset of first clinical symptom was 1.6 years old. Organ involvement was evaluated at the last hospital visit. Multi-organ involvement was diagnosed in all patients. Neurologic and GI symptoms were evident in all patients, and pulmonary and muscular involvement were common. Functional capacity of patients at their final visit during the study period was overall poor, with 51 patients (75%) bedridden and 15 patients (22%) expired. Forty-eight patients (70%) showed clinical regression during repeated hospital visits.

**Table 1:** Subject demographics (n = 68).

Patient characteristics	No. of patients (%)
Male: Female	29 (43): 39 (57)
Age at onset of first symptom, years	1.6 (0–15.5)*
Organ involvement (n, %)	
Central/Peripheral neurologic system	68 (100)
Gastrointestinal	68 (100)
Pulmonary	51 (75)
Muscular	35 (52)
Cardiac	11 (16)
Endocrinologic	9 (13)
Otologic	9 (13)
Ophthalmologic	8 (12)
Nephrologic	8 (12)
Haematologic	2 (3)
Functional capacity (n, %)	
Ambulatory	1 (1.5)
Wheelchair bound	1 (1.5)
Bedridden	51 (75)
Expired	15 (22)

*Data is given as mean value (range).

### Diagnostic evaluation of mitochondrial diseases

Patients went through multiple diagnostic studies for mitochondrial diseases **(**Table 2 **)**. Over half of patients showed non-specific features of mitochondrial diseases (52%). Thirty-three patients had syndromes compatible with mitochondrial diseases. Leigh’s syndrome was most common (41%), followed by mitochondrial encephalomyopathy, lactic acidosis, and stroke-like episodes (MELAS) (6%) and myoclonic epilepsy with ragged-red fibres (MERRF) (1%).

**Table 2:** Diagnostic evaluation of mitochondrial disease in study population (n = 68).

Diagnostic criteria	No. of patients (%)
Syndrome	
Leigh’s syndrome	28 (41)
MELAS	4 (6)
MERRF	1 (1)
Non-specific mitochondrial diseases	35 (52)
Lactic acidosis	39 (57)
Mitochondrial respiratory chain (MRC) defects (n = 60)	
MRC I	45/60 (75)
MRC II	1/60 (2)
MRC IV	5/60 (8)
Microscopic pathology (n = 62)	
Light microscopy	
Mitochondrial-disease specific	13/62 (21)
Nonspecific for mitochondrial disease	7/62 (11)
Electron microscopy	
Pleoconia	24/62 (39)
Megaconia	25/62 (40)

MELAS: mitochondrial encephalomyopathy, lactic acidosis, and stroke-like episodes.

MERRF: myoclonic epilepsy with ragged-red fibers.

Lactic acidosis was present in 39 patients (57%). Among 60 patients who underwent mitochondrial respiratory chain (MRC) complex evaluation, 51 patients had MRC defects (85%). MRC I defect was the most common manifestation (45 patients, 75%). Sixty-two patients underwent muscle biopsy analysed by microscopy; 13 patients (21%) showed ragged red fibres on light microscopy, a specific finding for mitochondrial diseases. Pleoconia and megaconia were evident in 24 and 25 patients each, respectively (39% and 40%).

### Gastrointestinal symptoms and indications for enteral tube feeding

Mean age of patients at the start of enteral tube feeding was 5.8 years, ranging from 0.5 to 18.5 years **(**Table 3 **)**. Mean duration from the first symptom of mitochondrial disease to enteral tube feeding was 4.6 years (range, 0–17.3 years). The majority of patients had GI symptoms requiring enteral tube feeding. The most common indication was oropharyngeal dysphagia, which was apparent in 55 patients (81%), followed by weight decrease in 43 patients (63%). Forty-nine patients were evaluated for gastroesophageal reflux (GER), among whom 36 underwent both oesophagography and 24-hour pH monitoring. While 39 of 49 patients had positive results in either study (80%), 24 hr pH monitoring rendered more positive results (33/39 examinees, 85%) than contrast oesophagography (18/46 examinees, 39%).

**Table 3:** Gastrointestinal symptoms and indications for enteral tube feeding (n = 68).

	No. of patients (%)
Age at the start of enteral tube feeding, years	5.8 (0.5–18.5)^a^
Time from the first symptom to enteral tube feeding, years	4.6 (0–17.3)^a^
Gastrointestinal symptoms (multiple indications)	
Oropharyngeal dysphagia	55 (81)
Weight decrease	43 (63)
Recurrent vomiting	42 (62)
Aspiration	40 (59)
Gastrointestinal haemorrhage	29 (43)
Regurgitation	22 (32)
Constipation	10 (15)
GER confirmed by imaging studies^b^	39/49 (80)
Contrast oesophagography	18/46 (39)
24 hr pH monitoring	33/39 (85)
Type of enteral tube feeding	
Gastrostomy	56 (82)
Prolonged nasogastric tube feeding	12 (18)

^a^Data is given as mean value (range).

^b^Out of 49 patients who went through any of the imaging evaluation for GER, 36 patients underwent both.

GER: gastroesophageal reflux.

As for the type of enteral tube feeding, 56 patients underwent surgical or percutaneous endoscopic gastrostomy (82%), and the other 12 patients were on prolonged nasogastric tube feeding (18%).

### Outcomes of enteral tube feeding

Effects of enteral tube feeding were analysed in patients with follow-up data available. Each outcome was compared among patients with NG tube versus gastrostomy **(**Table 4 **)**. Alleviation of GI symptoms was the most common effect of enteral tube feeding (37/48, 77%), evident in over 70% of both NG tube (6/7, 86%) and gastrostomy (31/41, 76%) patients.

**Table 4:** Outcomes of enteral tube feeding according to feeding type (n = 68) (No. of patients, %).

Enteral tube feeding	Decrease in GI symptoms (n = 48)	Weight gain (n = 64)	Increase in DQ (n = 48)
NG tube	6/7 (86)	1/10 (10)	1/4 (25)
Gastrostomy	31/41 (76)	17/54 (32)	9/44 (21)
Total	37/48 (77)	18/64 (28)	10/48 (21)

Decrease in GI symptoms: decreased symptoms for at least two consecutive hospital visits following the start of enteral tube feeding

Weight gain: increase in z-score in 1 year after the start of enteral tube feeding

Increase in DQ: increase of DQ after at least 3 months since the start of enteral tube feeding.

Weight gain was present in 18 of 65 patients (28%). The gastrostomy group had more patients with increased weight z-score after enteral tube feeding (17/55, 31%) compared to the NG tube group (1/10, 10%).

Increase in DQ was evident in 10 patients. No significant difference was found between NG tube (1/4, 25%) and gastrostomy (9/44, 21%) patients.

There was no statistically meaningful difference between the NG tube and gastrostomy groups regarding the three outcomes.

### Complications of enteral tube feeding

Forty-one of 68 patients (60%) had complications related to enteral tube feeding. (Table 5) Five of 12 NG tube patients (42%) had adverse effects, including haemorrhage related to NG tube placement (3/12, 25%) and mechanical tube problems (2/12, 17%). Thirty-six of 56 gastrostomy patients (64%) had complications, most commonly gastrostomy site granulation (19/56, 34%) and gastrostomy site leakage (10/56, 18%). Complications requiring removal of gastrostomy or additional gastrostomy occurred in 5 of 56 patients (9%). Two patients (4%) had morbid complications clinically related to gastrostomy.

**Table 5:** Complications of enteral tube feeding in paediatric patients with mitochondrial disease (n = 68).

Complications	No. of patients with NG tube (%, n = 12)	No. of patients with gastrostomy (%, n = 56)
Abdominal pain	1 (8)	1 (2)
Enteral tube-associated haemorrhage	3 (25)	5 (9)
Mechanical tube problems	2 (17)	3 (5)
Skin and mucosa alterations		
Ulcer	1 (8)	3 (5)
Infection	0 (0)	1 (2)
Gastrostomy site granulation	N/A	19 (34)
Gastrostomy site leakage	N/A	10 (18)
Complications requiring removal of gastrostomy or additional procedure/surgery	N/A	2 (4)
Morbidity related to gastrostomy	N/A	2 (4)

N/A: Not Available.

## Discussion

This study is the first survey of enteral tube feeding among paediatric patients with mitochondrial diseases. A few studies evaluating enteral tube feeding have included neuromuscular diseases^5,11,15–18^, but no study focused solely on mitochondrial diseases. As with a previous investigation including 73 patients^3^, mitochondrial disease patients in our study showed multi-organ involvement and severe clinical progress. As there is no cure available, supportive care is the mainstay of management in this patient group^2^. Thus GI support is needed as an important component of systemic management.

Our patients had multiple GI symptoms leading to enteral tube feeding. Previous studies pointed to swallowing difficulty or nutritional deficit resulting in weight loss as the major indication for PEG^5,7,17^. In our study, symptoms of swallowing difficulty including oropharyngeal dysphagia, vomiting, and aspiration, and decrease in weight occurred in the majority of patients leading to enteral tube feeding.

Outcomes of enteral tube feeding were decrease in GI symptoms, weight gain, and increase in DQ. In a study of patients with Rett syndrome, caregivers found decrease in vomiting or choking after gastrostomy^11^. In a study of 10 patients, decrease in GER was found after PEG insertion^19^. In our study, a significant percentage of patients in both the NG tube and gastrostomy groups showed a decrease in GI symptoms. Weight gain was prominent in both groups, with a higher percentage of patients showing weight gain in the gastrostomy group. Maintenance of weight gain has been reported in other studies investigating children with neuromuscular disorders, both with PEG and surgical gastrostomy^5,16^. The relative superiority of weight gain in the gastrostomy group is consistent with a recent study of 166 paediatric patients^9^, where fewer patients in the gastrostomy group remained underweight one year after tube feeding compared to the NG tube group. There was no statistical difference between the NG tube group and gastrostomy group in the two outcomes. As nutritional support is critical in the conservative care of incurable diseases and gastrostomy is relatively superior in providing nutritional improvement compared to NG tube, prompt gastrostomy could aid in the treatment of patients with mitochondrial diseases.

Developmental delay is a prominent feature of mitochondrial diseases, reported to be present in 68% to 85% of patients^3,4,20^. Yet there is no report on developmental outcomes as related to enteral tube feeding in this patient group. We noted positive short term outcomes with enteral tube feeding in terms of development. Long term studies would confirm whether enteral feeding could overcome the developmental regression reported to be present in 60% of patients with these diseases^21^.

The patients started enteral tube feeding 4.6 years in average after they had first symptoms of mitochondrial diseases. The majority of them had multiple GI symptoms, and we could not specify when each GI symptom appeared in this retrospective study. If the patients went through earlier evaluation and started enteral tube feeding before GI symptoms or growth failure aggravated, the results could have been more positive towards alleviation of GI symptoms, prevention of growth failure or deterioration of development.

A previously reported complication rate of NG tube was 46%^9^, consistent with our NG tube group (42%). The complication rate of gastrostomy feeding differs significantly among studies, ranging from 10%^8^ to 80%, with most complications being minor; 20% of patients reportedly develop major complications without morbidity^9^. Another recent study reported a 76% complication rate with 15% major complications^16^. In our study, 36 of 56 patients with gastrostomy (64%) had complications. While most patients had minor complications, there were 5 patients requiring removal of existing gastrostomy or additional procedure and 2 patients with morbidity related to the gastrostomy (13%). Despite severe disease progression and multi-organ involvement, the complication rate of our patients was compatible with previous studies on enteral tube feeding. As modification of feeding could efficiently enhance nutrition, gastrostomy could be a reasonable option for nutritional support in patients with mitochondrial diseases.

There are limitations to our study. This is a retrospective study with uncontrolled variables. We could not obtain details of the patients’ nutrition, including biochemical components and nutritional intake. The results are based on available data, which could not encompass all patients in this study, especially the outcomes and complications of enteral tube feeding. The outcomes and complications were evaluated on a numerical basis, and did not go through statistical analysis. Further statistical analysis could improve the power of the outcomes concerning enteral tube feeding among patient groups.

Nutrition is a crucial factor in supportive care of patients with mitochondrial disease. Enteral tube feeding could ameliorate growth failure and improve quality of life by relieving GI symptoms. Thus, the medical team should closely monitor and promptly manage symptoms indicating the need for enteral tube feeding as a part of systemic management.
